# Leisure Time Activities and Subjective Happiness in Early Adolescents from Three Ibero-American Countries: The Cases of Brazil, Chile and Spain

**DOI:** 10.3390/children10061058

**Published:** 2023-06-14

**Authors:** Diego Gomez-Baya, Tania Gaspar, Rafael Corrêa, Javier Augusto Nicoletti, Francisco Jose Garcia-Moro

**Affiliations:** 1 Department of Social, Developmental and Educational Psychology, Universidad de Huelva, 21071 Huelva, Spain; fjose.garcia@dpsi.uhu.es; 2 Environmental Health Institute, Universidade de Lisboa, 1649-028 Lisboa, Portugal; tania.gaspar.barra@gmail.com; 3 School of Psychology and Life Sciences, Universidade Lusofona, 1749-024 Lisboa, Portugal; 4 School of Public Policy and Government, Getúlio Vargas Foundation, Rio de Janeiro 22231-010, Brazil; rafael.correa@fgv.br; 5 Department of Humanities and Social Sciences, Universidad Nacional de La Matanza, Buenos Aires B1754JEC, Argentina; javiernicoletti@gmail.com

**Keywords:** leisure time activities, subjective happiness, early adolescents, cross-sectional, Ibero-American

## Abstract

(1) Background: The aim of the present study is to analyze subjective happiness in relation to leisure time in 10-year-old boys and girls from Brazil, Chile and Spain and to determine which leisure time activity has a greater effect on their subjective happiness and whether there are differences according to gender. (2) Methods: Data from the third wave of the Children’s Worlds Project was used, which was collected by administering self-report measures to representative samples in each country. The sample was composed of 4008 early adolescents from Brazil (22.1%, *n* = 886), Chile (22.8%, *n* = 913) and Spain (55.1%, *n* = 2209). The mean age of the sample was 10.17 years (SD = 0.57), and 51.7% were girls. (3) Results: In terms of subjective happiness, moderate to high means were observed, with higher scores in boys and the Spanish sample. The results showed some differences in leisure time activities among children from Brazil, Chile and Spain. Furthermore, the results highlighted the importance of relaxation time with the family to promote happiness in pre-adolescence across genders and countries. Additionally, playing outdoors and doing homework were also found to be protective factors for subjective happiness. (4) Conclusions: In Brazil, Chile and Spain the most consistent result was the positive effect of spending time relaxing with the family on subjective happiness.

## 1. Introduction

The promotion of child health and psychological well-being is an important goal around the world and should be nurtured across the lifespan [1,2]. In this sense, the importance of leisure time activities as a key element to fostering positive development has been examined in a growing number of studies [3,4,5,6,7,8]. 

The type of activities performed during leisure time by children has become a topic of special scientific and social relevance because of their consequences for present and future psychological well-being [8,9,10]. Larson and Verma [9] examined the change in time use among children and adolescents around the world and discussed its developmental consequences. These authors observed that adolescents in East Asian postindustrial societies spent their free time doing homework, which was related to lower intrinsic motivation but higher achievement. Furthermore, adolescents in North America spent more time in leisure activities, which had more self-direction but did not show a clear effect on concrete developmental outcomes. McHale et al. [10] analyzed the relationships between free-time activities in middle childhood and adjustment in early adolescence. These authors examined several free-time activities, i.e., hobbies, sports, toys and games, outdoor play, reading, television viewing, and hanging out, and their relationships, after a 2-year follow-up, with school grades, conduct problems, and depression symptoms. They concluded that more structured activities, such as practicing hobbies and sports, were the most development-enhancing ways for children to spend free time, compared to hanging out, which was related to poorer school grades and more conduct problems. 

A recent study by Anh and Yoo [8] analyzed cross-sectional data about the patterns of time use and life satisfaction in a sample of 12-year-old adolescents from 18 countries (i.e., Algeria, Nepal, Estonia, Spain, Columbia, Turkey, Ethiopia, South Korea, Germany, UK, Israel, Romania, Norway, Poland, South Africa, Malta, Finland and Italy), collected in 2013–2015. This work examined a wide range of free-time activities, i.e., taking classes outside school times on matters different than at school, participating in organized leisure time activities, reading for fun (not homework), helping around the house, doing homework, watching TV or listening to music, playing sports or doing exercise, using a computer and taking care of family members. The results pointed out that the activities children most frequently engage in are doing homework and watching TV, in both boys and girls. However, boys reported more sport practice, and girls indicated more frequent help with housework. Concerning the associations with life satisfaction, the authors concluded that engagement with a diverse range of activities was protective. Lower scores in life satisfaction were observed in girls with more frequent engagement in housework and in boys with greater screen time.

Leisure time activities may influence child and adolescent well-being because they provide different opportunities for positive experiences. Children and adolescents may evaluate the quality of these experiences, and those assessments may affect their perceptions about their own lives. In this line, the concept of subjective happiness has been widely studied in psychology, which refers to the subjective self-evaluation that a person makes of his or her overall life [11]. The psychology of subjective happiness comprises the scientific analysis of how people evaluate their own lives in specific life areas or in an overall way [2]. This self-assessment has been analyzed as a positive indicator of psychological adjustment in adolescent samples, associated with positive developmental outcomes. When examining the impact of different developmental contexts on subjective happiness, children reported that the most important areas were family climate, leisure time, friendship, school adjustment and receiving gifts [12]. Thus, more research is necessary about leisure as a key element for adolescent development [8], since leisure time may provide many pleasant experiences to enhance subjective happiness. With regards to this effect on happiness, leisure time activities may differ in the type of emotions, motivations, values, benefits and perceptions of adolescents [13,14].

Moreover, the adolescent use of free time is highly influenced by family and community characteristics as well as sociocultural aspects [8,15]. These contextual characteristics also have direct effects on both physical and mental health, with social and emotional competencies playing a key mediational role [16]. The daily activities in which children spend their free time may encourage or hinder the development of skills, knowledge, and lifestyles that in turn impact their psychological well-being [8]. Research to date has indicated that how children spend their free time has differential consequences for psychological well-being, so sharing or spending time with others, i.e., family or friends, increases feelings of happiness. Specifically, the amount and quality of time spent with others and the frequency of acts of kindness contribute to greater happiness than free-time behaviors aimed at obtaining only material goals [17,18]. 

In the last decade, the use of digital media has become the most popular leisure activity among children and adolescents. The effects of digital media use on children’s brains and cognitive processes have been widely debated [19]. Many studies have concluded that spending many hours in front of a screen negatively influences children’s physical and mental health [20,21,22]. Furthermore, although free time may constitute a source of personal satisfaction [12], time spent by adolescents in unstructured activities with friends without adult supervision is considered a risk factor for engaging in antisocial behavior [23]. Contrarily, some studies have underlined that warm affective bonds within the family and positive peer relationships are fundamental sources of perceived well-being [24,25].

Despite the importance of children’s use of leisure time for their perceived well-being, more international comparative studies are needed [8]. Further research is also needed on the impact of different types of leisure activities on subjective happiness. More research is highly recommended to collect evidence to guide program design aimed at preventing situations that hinder positive child development and promoting leisure activities that foster well-being. Most research to date has been conducted in Anglo-Saxon countries, while more evidence is required in Ibero-American countries. 

The present research aims to provide evidence in this sense by comparing representative samples of boys and girls from Brazil, Chile and Spain, aged 10 years old—just before the academic transition to secondary education. This period is especially important for the beginning and development of healthy lifestyles across the lifespan. These three Ibero-American countries share a common cultural and historical background, which may influence lifestyles and perceptions of well-being. This research aims to contribute to the development of evidence-based public policies that promote active leisure activities in the free time that favor subjective happiness in school-age children in Brazil, Chile, and Spain [26]. Thus, the general aims of the present exploratory study are to analyze leisure time activities and their associations with subjective happiness in 10-year-old girls and boys from these three Ibero-American countries.

## 2. Materials and Methods

### 2.1. Procedure Collection Procedure and Sample

The data used in this publication come from the third wave of the Children’s Worlds Project: An international survey of children’s lives and well-being (www.isciweb.org, accessed on 1 May 2023). A database of 10-year-old adolescents from Brazil, Chile and Spain was used for the purposes of the present study. These countries were selected due to similarities in culture and language. Brazil, Chile and Spain were the three Ibero-American countries participating in the international project. Three cross-sectional studies were performed in each country during the period of 2016–2019. Self-report measures were administered to a representative sample in each country. A sample size of around 1000 participants was recruited in each country, although just around 400 students were required with a confidence level of 95% and a margin of error of 5%. The Children’s Worlds project began in 2009, founded by a group of researchers from the International Society for Child Indicators who discussed in a meeting hosted by UNICEF the need for an international survey about children’s lives and daily activities, their time use and their own perceptions and evaluations of their well-being. In that meeting, a draft version of the questionnaire was designed. These self-report measures were well translated, tested, and validated in Spanish (specific versions for Chile and Spain) and Portuguese (version for Brazil) in 2010–2011, and were used in the two previous waves of the international study (in 2011–2012 and in 2013–2014, respectively). National reports and publications have well documented the quality of the instruments in the three waves of research. Methodological details for each separate country in the third wave were described in more detail in: https://isciweb.org/wp-content/uploads/2020/08/Childrens-Worlds-3rd-Wave-National-Reports-with-England.pdf (accessed on 1 May 2023). 

The sample was composed of 4008 early adolescents from Brazil (22.1%, *n* = 886), Chile (22.8%, *n* = 913) and Spain (55.1%, *n* = 2209). The mean age of the sample was 10.17 (SD = 0.57) and 51.7% were girls. Most of the sample always slept in the same home (98.9%) and lived with their families (97.7%).

### 2.2. Instrument

Subjective happiness. A Likert-type item was used to assess subjective happiness. The indicator was introduced by the sentence “I am happy with my life”, and 11 response options were presented, from ‘Not at all agree’ to ‘Totally agree’.

For leisure time activities, 10 activities were selected to examine leisure time activities in early adolescence. After the question “How often do you spend time?”, these activities were presented: helping around the house, taking care of siblings or others, doing extra classes/tuition, doing homework/studying, watching TV, playing sports/doing exercise, relaxing with family, playing/time outside, using social media, and playing electronic games. Six response options were presented: never, less than once a week, once or twice a week, three or four days a week, five or six days a week, and every day. This scale showed acceptable internal consistency and reliability (α = 0.62), considering the wide range of activities included.

### 2.3. Data Analysis Design

First, the frequency distribution of leisure time activities was examined, as were descriptive statistics for subjective happiness. Differences by gender and country were examined in leisure time activities by conducting Chi-squared tests. Concerning differences by gender and country in subjective happiness, a *t*-test and variance analysis were performed, respectively. Second, linear regression analyses were developed to explain subjective happiness based on the ten leisure time activities, controlling for gender and country. Adjusted R-squared and standardized coefficients were examined. After these regression analyses, the activity with the highest effect on subjective happiness was separately studied by performing a variance analysis controlling for gender and country. 

## 3. Results

### 3.1. Descriptive Statistics

Table 1 and Table 2 show the frequency distribution of leisure time activities by country and gender, respectively. In general, the activities with the highest frequencies were watching TV and relaxing time with family, with more than half of the sample spending time with them every day. Furthermore, the activities with the lowest frequencies were doing extra classes and taking care of siblings or others. First, some significant differences were observed by country. The greatest frequency of helping around the house was observed in Brazil, χ2(10) = 116.47, *p* < 0.001, φ = 0.174, while the Spanish preadolescents spent more time taking care of siblings or others, χ2(10) = 108.92, *p* < 0.001, φ = 0.169. In the Brazilian subsample, the greatest frequency of doing extra classes was observed, χ2(10) = 168.62, *p* < 0.001, φ = 0.211, while the Spanish subsample spent more time doing homework or studying, χ2(10) = 258.14, *p* < 0.001, φ = 0.261. Concerning watching TV, Brazilian preadolescents reached the greatest frequency, χ2(10) = 75.64, *p* < 0.001, φ = 0.141. Chilean participants played sports or exercised with more frequency than those from Spain and Brazil, χ2(10) = 298.99, *p* < 0.001, φ = 0.280. Furthermore, Brazilian participants spent less time relaxing with family, χ2(10) = 77.58, *p* < 0.001, φ = 0.144, but spent more time playing outside, χ2(10) = 130.77, *p* < 0.001, φ = 0.186. Brazilian preadolescents also spent more time using social media, χ2(10) = 188.86, *p* < 0.001, φ = 0.223, and playing electronic games, χ2(10) = 209.39, *p* < 0.001, φ = 0.234, with the lowest percentages in the Spanish sample.

Second, some gender differences were also detected. Girls spent more time helping around the house, χ2(5) = 31.41, *p* < 0.001, φ = 0.091, doing homework, χ2(5) = 38.01, *p* < 0.001, φ = 0.101, relaxing with family, χ2(5) = 11.59, *p* = 0.041, φ = 0.056, and using social media, χ2(5) = 14.56, *p* = 0.012, φ = 0.062. Boys spent more time watching tv, χ2(5) = 24.42, *p* < 0.001, φ = 0.080, playing sports, χ2(5) = 61.75, *p* < 0.001, φ = 0.128, playing outside, χ2(5) = 13.96, *p* = 0.016, φ = 0.061, and playing electronic games, χ2(5) = 339.63, *p* < 0.001, φ = 0.299. No differences were found in taking care of siblings or doing extra classes. 

**Figure 1:**
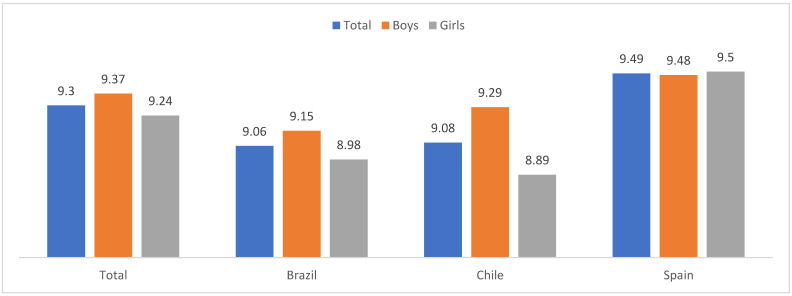
Means in subjective happiness by gender and country.

Figure 1 presents the means of subjective happiness by gender and country. In general, a moderate to high mean score was found. Gender differences were found, with higher means in boys, *t*(3895) = 2.21, *p* = 0.027, d = 0.074. Country differences were also observed, with more happiness among Spanish participants, F(2, 3932) = 27.53, *p* < 0.001, ηp2 = 0.014. Gender and country had a significant interaction to explain subjective happiness, F(2, 3991) = 4.67, *p* = 0.009, ηp2 = 0.002, with the highest means in Spanish girls and the lowest ones in Chilean girls. Gender differences were only significant in the Chilean sample, *t*(866) = 2.87, *p* = 0.004, d = 0.193.

### 3.2. Linear Regression Analyses

Table 3 describes the results of linear regression analyses to explain subjective happiness, controlling for gender and country. In the total sample, positive effects were observed by relaxing with family, playing outside, and doing homework/studying, and a negative effect was found by taking extra classes. The greatest positive effect was observed by relaxing with family, which was consistent across genders and countries. In boys, a negative effect was observed by using social media, while in girls, that negative effect was detected by playing electronic games. In girls, helping around the house had a small positive effect, while in boys, doing extra classes did not have a significant effect. 

Comparing the results by country, in Brazilian preadolescents, relaxing with family and watching TV had positive effects, while doing extra classes and using social media had negative ones. In Chile, the unique significant predictor was relaxing with family, with the greatest standardized coefficient. In Spain, doing extra classes and playing electronic games had negative effects, while relaxing with family, doing homework, and playing outside showed positive effects on happiness. When examining the percentage of explained variance by leisure time activities, the highest R2 was observed in the subsample of girls and the subsample from Chile.

**Figure 2:**
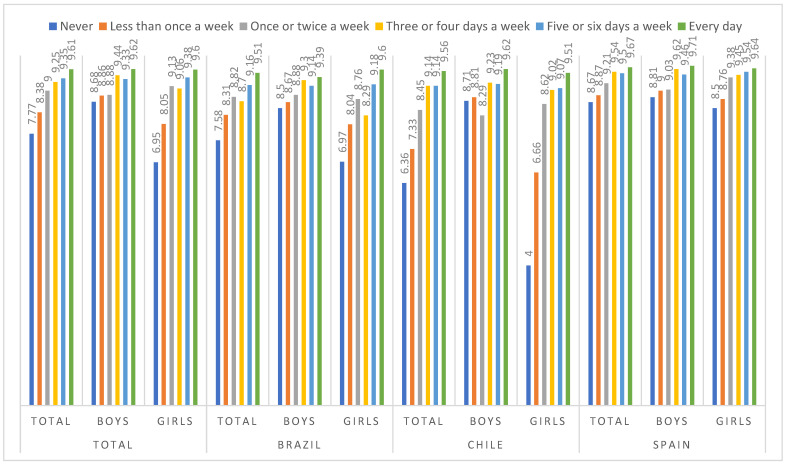
Means in subjective happiness, by the categories of the indicator of relaxing with family, gender and country.

After these results, relaxing time with family was the most important leisure activity for subjective happiness in the sample examined. Thus, a variance analysis was conducted to examine subjective happiness based on the frequency of relaxing with family, controlling for gender and country. Figure 2 shows the means of subjective happiness by the categories of the indicator of relaxing with family and by gender and country. A significant interaction was observed between relaxing time with family × gender × country, F(10, 3627) = 4.78, *p* < 0.001, ηp2 = 0.013. The lowest subjective happiness was observed in Chilean girls who spent less time a week relating to family (M = 4.00, SD = 4.02), while the greatest subjective happiness was observed in Spanish boys who spent time relaxing with family every day (M = 9.71, SD = 1.16). 

## 4. Discussion

The aim of the present study was to examine the relationships between leisure time activities and subjective happiness in Brazil, Chile and Spain and to compare these relationships by gender. This work has highlighted some interesting findings. The leisure time activities with the highest daily frequency were “watching television” and “relaxing with the family”, with percentages above 50%. Compared to boys, girls showed higher frequency in “helping at home”, “doing homework/studying” and “using social networks”. Boys spent more time watching TV, playing sports, playing outdoors and playing electronic games than girls. In addition, it was observed that the Brazilian sample spent more time watching television, using social networks and playing electronic games; the Spanish sample spent more time doing homework and the Chilean sample practiced sports more frequently. Regarding subjective happiness, moderate to high means were observed, with higher scores in the boys’ sample and the Spanish sample.

In line with biopsychosocial perspectives that emphasize the importance of sociocultural and contextual variables as influencing elements of well-being [27,28], one remarkable result was observed concerning the effect of leisure time on well-being. In all three countries, the most consistent result was the positive effect of spending time relaxing with the family, although it is true that this was more pronounced in Chile. This aspect is especially significant considering that relationships within the family influence the cognitive and affective aspects of happiness reported by children [29,30,31], especially when affective climate, understanding and dialogue are provided by family members [32].

In addition to the importance of spending time with family, the results also highlighted the importance of playing outdoors and doing homework as positive elements to enhance subjective happiness.

Although few gender differences in subjective well-being are usually observed in child samples, some differences in the relationships with leisure time activities were detected [33]. Thus, the data obtained in the present research indicated that, in girls, “doing extra classes” and “playing electronic games” had a negative effect on well-being, whereas, in boys, this negative effect was observed in “using social networks”. The results indicated that the lowest score in subjective happiness was found in Chilean girls with a lower frequency of “family relaxation time”, while the highest subjective happiness was reported by Spanish boys with “daily family relaxation time”. This result is consistent with previous research on this topic [34].

Despite the interesting contributions provided, some limitations should be recognized. Due to the cross-sectional design, conclusions can only be based on bidirectional associations. As a future research line, a longitudinal study is recommended to explore the directionality of the relationships between leisure time activities and subjective happiness. Moreover, since the data were collected through the application of a self-report questionnaire, only subjective information was examined. Therefore, future research could explore leisure time activities with more objective measures and use multiple informants. In addition, it could be important to assess not only the times per week but also the number of hours per day and the quality of those leisure time activities. Specifically, concerning playing and sport practice, further examination in future research is needed to examine different types of physical exercise in leisure time, as well as how some characteristics may be more associated with happiness (i.e., competitive, recreation, at the weekend or daily, in nature, etc.). Finally, future work could examine other variables, such as measures of psychological symptoms and the quality of family relationships and the community context. In this line, family structure should also be controlled in future research, as should the number, sex, age and position of siblings, which may vary in the three countries. Along these lines, the framework of developmental assets, with both internal and external resources, could be promising for reaching a better understanding of the mechanisms through which leisure time may influence well-being. Further research could also address the possible socioeconomic differences in leisure time and well-being, as already noted by Auhuber et al. [35]. 

The results pointed out the need to understand adolescent happiness from an ecological perspective. This work underlines the importance of individual factors, i.e., gender, since different leisure activities were found to contribute to well-being differently in boys and girls. Active or sedentary leisure time activities can be differently related to the perception of happiness by gender. Social relationships, especially the relationship with the family, as well as interactions with other developmental contexts, such as school and extracurricular activities in the community, may also explain adolescents’ happiness. Furthermore, culture and parental values may also influence adolescents’ perceptions of happiness. Thus, in the three countries involved in the study, the common protective factor found was spending positive time with the family, but specificities were also identified in each country that should be considered when designing public policies to promote happiness and leisure activities. The results of the present study may suggest some possible practical implications. Leisure time activities may provide the opportunity to build resources for child well-being and improve family connectedness. In this line, the intervention performed by Pluta et al. [36] concluded that both quantity and quality of leisure time were important for family well-being. This intervention showed that the practice of physical activity and playing with family members had a positive effect on children’s well-being. As suggested by Schwab and Dustin [37], leisure time spent with family may constitute an opportunity to develop problem-solving skills, enhance compromise and bonding and in turn improve family functioning and well-being. Furthermore, leisure time activities may also take gender differences and the promotion of equal opportunities for well-being into account. Consequently, the design of interventions should address gender differences in parental engagement in leisure time [38] and differential factors influencing motivation in boys and girls [39]. Thus, Ibero-American policies to promote child well-being should consider the importance of leisure time, specifically active leisure time with family and the differential factors influencing girls and boys. 

## 5. Conclusions

In conclusion, the present study showed some differences in leisure time and subjective happiness in girls and boys from Brazil, Chile and Spain and highlighted the importance of relaxing time with the family to promote happiness in preadolescence. This result underlines the importance of effective relationships within the family, despite the changes in leisure activities because of the rise of new technologies, which may foster sedentary and isolated behavior. The results underline the need to design psychosocial programs to promote healthy opportunities for children and their families to enjoy leisure time. More resources should be provided to families in their communities to spend time together, specifically more options for practicing safe play and exercise. A systemic view of child well-being, conceived as a result of family dynamics, could increase attention towards developing parenting practices that encourage joint and healthy leisure time experiences in order to satisfy the needs of bonding and play in childhood. 

## Figures and Tables

**Table 1:** Frequency distribution of leisure time activities by country.

		Never	Less than Once a Week	Once or Twice a Week	Three or Four Days a Week	Five or Six Days a Week	Every Day
Helping around the house	Brazil	9.1	15.8	16.8	9.1	11	38.2
	Chile	6.6	10.1	18.3	18.6	14.9	31.5
	Spain	3.3	11.7	21.5	19.3	12.2	32.1
Taking care of siblings or others	Brazil	39.8	11.8	8	6.6	4.7	29.1
	Chile	33.4	11.1	8.9	6.4	8.3	31.8
	Spain	23.7	9.3	10.4	8.6	9.1	39
Doing extra classes/tuition	Brazil	67.5	6.3	9.6	4.7	2	10
	Chile	80	5.9	4.6	2.6	2.8	4
	Spain	61.3	5.7	18.7	3.7	3.3	7.3
Doing homework/studying	Brazil	7	10.8	8.8	10.5	12.6	50.3
	Chile	6.3	7.2	15	16.3	18.9	36.2
	Spain	1.5	2.8	9.3	12.5	13.8	60.2
Watching TV	Brazil	4.6	7.7	6.9	6.5	8.9	65.4
	Chile	3.8	5.2	7.8	11.8	13.1	58.3
	Spain	2.6	5.1	11.3	12.5	13.8	54.8
Playing sports/doing exercise	Brazil	18.6	13.3	17.2	12	8.9	29.9
	Chile	8.9	6.3	11.4	16.3	17.2	40
	Spain	4.7	5.3	22	21.8	12.7	33.5
Relaxing with family	Brazil	7.2	11	11.1	9.8	10.4	50.4
	Chile	4	6.3	7.7	12.3	16.3	53.4
	Spain	2.9	6.3	12.5	10.2	16	52.1
Playing/time outside	Brazil	10.4	14	11	10.7	12.7	41.3
	Chile	10.5	12.4	13.6	15	15.1	33.4
	Spain	3.4	8.4	15.6	14.6	18.4	39.7
Using social media	Brazil	16.7	7.8	7	7.8	6.7	53.9
	Chile	12.5	6.7	9.1	10.6	15.7	45.4
	Spain	16.7	10.1	14.5	13.3	13.9	31.4
Playing electronic games	Brazil	12.5	8.3	8.7	8.2	10	52.4
	Chile	10.5	8.4	11.8	11.8	14.6	42.9
	Spain	14.1	13	18.9	14.3	11.5	28.2

**Table 2:** Frequency distribution of leisure time activities by gender.

		Never	Less than Once a Week	Once or Twice a Week	Three or Four Days a Week	Five or Six Days a Week	Every Day
Helping around the house	Total	5.4	12.2	19.7	16.8	12.5	33.4
	Boys	7	12.4	18.6	18.1	13	30.9
	Girls	3.8	12.1	20.6	15.4	12.2	35.8
Taking care of siblings or others	Total	29.6	10.3	9.5	7.6	7.9	35
	Boys	29.4	9.8	9.1	8.4	8.7	34.5
	Girls	29.8	10.8	9.8	6.9	7.2	35.5
Doing extra classes/tuition	Total	67.1	5.9	13.3	3.7	2.9	7.1
	Boys	66.4	5.9	13.5	3.5	3.1	7.6
	Girls	67.5	5.8	13.3	3.9	2.7	6.8
Doing homework/studying	Total	3.9	5.6	10.5	12.9	14.7	52.3
	Boys	5.1	6.3	11.5	14	15.2	47.9
	Girls	2.7	5	9.7	11.7	14.3	56.6
Watching TV	Total	3.3	5.7	9.4	11	12.5	58
	Boys	2.9	4.4	8.2	10.6	12.5	61.3
	Girls	3.8	6.9	10.5	11.4	12.5	55
Playing sports/doing exercise	Total	8.8	7.3	18.4	18.3	12.9	34.2
	Boys	7.5	5.4	16.1	18.5	12.9	39.5
	Girls	9.9	9.1	20.6	18.1	12.8	29.4
Relaxing with family	Total	4.1	7.4	11.1	10.6	14.8	52
	Boys	4.1	6.4	11.5	11.3	16	50.6
	Girls	4.1	8.2	10.5	10.1	13.6	53.5
Playing/time outside	Total	6.6	10.6	14.1	13.8	16.3	38.6
	Boys	6.5	9.1	12.9	15	16.9	39.6
	Girls	6.7	11.7	15.1	12.6	15.9	38
Using social media	Total	15.7	8.8	11.5	11.4	12.6	39.8
	Boys	17.4	7.8	10.9	12.1	12	39.9
	Girls	14.3	9.9	12.1	10.7	13.2	39.8
Playing electronic games	Total	12.9	10.9	14.9	12.3	11.9	37.1
	Boys	5.3	5.8	14.8	15.4	12.9	45.8
	Girls	20.1	15.5	14.8	9.5	11	29.1

**Table 3:** Linear regression analyses to explain subjective happiness based on the leisure time by gender and country.

	Total F = 29.71 *** R^2^ = 0.096		Boys F = 9.06 *** R^2^ = 0.054		Girls F = 24.55 *** R^2^ = 0.133		Brazil F = 8.55 *** R^2^ = 0.097		Chile F = 13.21 *** R^2^ = 0.149		Spain F = 9.83 *** R^2^ = 0.054	
	*t*	β	*t*	β	*t*	β	*t*	β	*t*	β	*t*	β
Country	4.83	0.09 ***	3.18	0.08 **	3.58	0.09 ***						
Gender	−2.89	−0.05 **					−0.69	−0.03	−2.56	−0.09 *	−1.99	−0.05 *
Helping around the house	1.87	−0.04	0.41	0.01	2.33	0.06 *	−0.04	−0.01	1.88	0.07	1.40	0.04
Taking care of siblings or others	−1.83	−0.03	−0.50	−0.01	−1.94	−0.05	−0.97	−0.04	−1.75	−0.06	−0.72	−0.02
Doing extra classes/tuition	−3.52	−0.06 ***	−1.77	−0.05	−3.00	−0.07 **	−2.04	−0.07 *	−0.80	−0.03	−3.02	−0.07 **
Doing homework/studying	3.45	0.06 **	2.31	0.06*	2.75	0.07 **	0.41	0.02	1.02	0.04	3.81	0.10 ***
Watching TV	1.59	0.03	0.84	0.02	1.60	0.04	4.05	0.15 ***	−0.73	−0.03	−1.13	−0.03
Playing sports/doing exercise	1.64	0.03	0.39	0.01	1.74	0.04	0.45	0.02	1.59	0.06	0.92	0.02
Relaxing with family	10.18	0.20 ***	4.32	0.13 ***	9.54	0.25 ***	5.16	0.22 ***	7.64	0.31 ***	5.46	0.15 ***
Playing/time outside	4.11	0.08 ***	3.21	0.10 **	2.70	0.07 **	1.52	0.06	1.35	0.06	2.67	0.07 **
Using social media	−1.43	−0.03	−2.10	−0.06 *	0.22	0.01	−2.35	−0.09 *	−0.76	−0.03	0.88	0.02
Playing electronic games	−1.62	−0.03	0.87	0.03	−2.79	−0.08 **	−0.74	−0.03	0.37	0.02	−1.98	−0.06 *

Note. *** *p* < 0.001, ** *p* < 0.01, * *p* < 0.05.

## Data Availability

Data are available on the webpage of the Children’s Worlds project: An international survey of children’s lives and well-being (www.isciweb.org, accessed on 1 April 2023).
